# Lay-Up Compound Matrices for Application of Medical Protective Clothing: Manufacturing Techniques and Property Evaluations

**DOI:** 10.3390/polym14061179

**Published:** 2022-03-16

**Authors:** Ching-Wen Lou, Jian-Hong Lin, Mei-Feng Lai, Chen-Hung Huang, Bing-Chiuan Shiu, Jia-Horng Lin

**Affiliations:** 1 Fujian Key Laboratory of Novel Functional Fibers and Materials, Minjiang University, Fuzhou 350108, China; cwlou@asia.edu.tw; 2 Advanced Medical Care and Protection Technology Research Center, College of Textile and Clothing, Qingdao University, Qingdao 266071, China; 3 Department of Bioinformatics and Medical Engineering, Asia University, Taichung City 413305, Taiwan; 4 Department of Medical Research, China Medical University Hospital, China Medical University, Taichung City 404333, Taiwan; 5 Laboratory of Fiber Application and Manufacturing, Department of Fiber and Composite Materials, Feng Chia University, Taichung 40724, Taiwan; baron.lin69@gmail.com; 6 Department of Aerospace and Systems Engineering, Feng Chia University, Taichung City 40724, Taiwan; 7 College of Material and Chemical Engineering, Minjiang University, Fuzhou 350108, China; bcshiu@mju.edu.cn; 8 School of Chinese Medicine, China Medical University, Taichung City 404333, Taiwan; 9 Advanced Medical Care and Protection Technology Research Center, Department of Fiber and Composite Materials, Feng Chia University, Taichung City 407102, Taiwan

**Keywords:** Tencel fibers, recycled Kevlar fibers, electrostatic spinning, breathable and waterproof membranes, lay-up compound technique

## Abstract

Medical protective clothing is the first line of defense for medical staff, which makes the acquisition of protection and multiple function challenging. When it comes to contagious diseases, the physical properties of protective clothing are deemed the top priority and, subsequently, they have significant meaning for the structural design, production cost evaluation, convenient production, and innovation. In this study, nonwoven technology is employed to produce matrices in which mechanical properties are supported by Tencel fibers and recycled Kevlar fibers. Next, the electrostatic spinning is conducted to generate breathable and waterproof films. The nonwoven fabrics and membranes are combined to have diverse functions, forming lay-up compound matrices for medical protective clothing. Moreover, measurements are conducted to characterize the lay-up compound matrices in terms of the tensile strength, tearing strength, bursting strength, puncture resistance, stiffness, air-permeable property, surface resistance, comfort performance, sub-micron particulate filtration efficiency, and the penetration of synthetic blood. As for the nonwoven fabrics, the mechanical properties are significantly improved after Kevlar fibers are incorporated. The tensile strength is (62.6 ± 2.4) N along the machine direction (MD) and (50.1 ± 3.1) N along the cross machine direction (CD); the tearing strength is (29.5 ± 1.6) N along the MD and (43.0 ± 1.7) N along the CD; the bursting strength is (365.8 ± 5.0) kPa; and the puncture resistance is (22.6 ± 1.0) N. Moreover, the lay-up compound matrices exhibit a stiffness of (14.7 ± 0.2) cm along the MD and (14.6 ± 0.1) cm along the CD, a surface resistance of (2.85 × 10^9^ ± 0.37 × 10^9^) Ω, an air-permeable property of (45.4 ± 2.3) cm^3^/s/cm^2^, and sub-micron particulate filtration efficiency of over 98%. In the measurement for penetration of synthetic blood, the K40/PAN/TPU group prevents the synthetic blood from penetration. Hence, the incorporation of recycled Kevlar fibers and lay-up compound technique creates good physical properties, an appropriate comfort attribute, and functions, which suggests that this study provides a greater diversity and new concepts for the production of medical protective clothing.

## 1. Introduction

With the constant progress made by studies on contagious viruses, e.g., human immunodeficiency virus (HIV), hepatitis B virus (HBV), and hepatitis C virus (HCV), it is critical to maintain life safety and the health of the frontline workers via protection, control, correspondence, and investigation. Specifically, prevention is the most effective measure to block the spread of contagious viruses, and, therefore, advancement of suitable protective clothing becomes the priority [1,2]. Serving as an important barrier against the cross infection between patients and medical staff, medical protective clothing has been extensively studied. Medical protective clothing is particularly used by medical staff (e.g., doctors, nurses, public health staff, janitors) and people who enter a specified area (e.g., patients, visitors, workers in infection area), and its main function is to improve the medical hygiene environment and protect medical staff. Used not only in hospitals but also in clinics, laboratories, and first-aid stations, medical protective clothing becomes one popular item for protective clothing studies [3].

Protective clothing is mainly to protect the safety and health of the users and can provide physical, chemical, nuclear, cold/hold environment, or pathogen protection [4]. Physical/chemical protection prevents the users from the cut of knives or the penetration of bullets, oil, water, or corrosive liquids [5,6,7]. Thermal protection prevents or reduces the damage caused by radiant heat, flame, hot air, molten metal substances, steam, and hot liquid splashes [8,9,10]. As for protective clothing against pathogens, e.g., microbes, bacteria, and virus, the related studies only focused on the introduction of materials and garment development, so the empirical studies did not start until the outbreak of SARS. Afterwards, only a few scholars conducted further explorations on innovative materials and manufacturing process [4,11,12,13,14,15,16,17,18]. In the wake of COVID-19, the continuous progress of medical protective clothing appears even more important.

Medical protective clothing and textiles are usually composed of woven fabric, nonwoven fabrics, and composites, and can be specifically divided into: (a) cotton protective clothing, (b) nonwoven protective clothing, (c) high-density polyurethane coating protective clothing, and (d) multi-layered protective clothing. Cotton protective clothing demonstrates common protection and is suitable for a dry environment. However, it shows an awful microbial barrier effect and is accompanied by the presence of cotton flocks. By contrast, made by nonwoven technology (e.g., needle-bonded, spunlace, and meltblown), nonwoven protective clothing demonstrates better dry/wet status protection, whereas it also has a low resistance to a hydrostatic pressure and a poor filter efficacy against particles. By comparison, coating protective clothing demonstrates an improved hydrostatic pressure and greater filter efficacy against particles than the nonwoven one, but it also has disadvantages of peeling off while aging, as well as a low air-permeable property. Finally, multi-layered protective clothing with improved performances is developed, and it also shows excellent comfort and strengths, but the production cost is considerably high [14,19,20,21,22].

Therefore, multi-layered protective clothing is the main frame of this study that particularly aims to develop medical protective clothing with efficient methods and the required properties. The nonwoven technology is employed to produce protective clothing matrices. Tencel fibers are the main material in this experiment and possess high strength, high purity, high moisture absorption, and fluffy/draping properties that are ascribed to the crimped attribute. As a result, the use of Tencel fibers provides fabric matrices with a good texture [23]. Nonetheless, Tencel fibers do not have standard mechanical strengths required by medical protective clothing, and it is thus required to have other reinforcing materials [24]. In this case, Kevlar fibers are used as reinforcement for Tencel fibers because Kevlar fibers exhibit high strength, light mass, high stability, and excellent chemical resistance that compensate for the downsides of Tencel fibers. Concurrently, low melting point polyester (LMPET) fibers are used to bond and reinforce Tencel and Kevlar fibers [25]. The composites are then combined with breathable and waterproof membranes, thereby providing the final product with functions as required by comprehensive medical protective clothing. The proposed matrices for protection gear are no longer mono-functional. In addition to the mechanical strength that conventional protection gear requires, the materials embody comfortable, dust-proof, and synthetic blood-proof features. In general, most protection gear fails to present all requirements at the same time, e.g., the acquisition of basic protection at a cost of absence of pollution-proof attribute, or the provision of a light weight and pollution resistance instead of physical protection. Hence, the main purpose of this study is to develop matrices of medical protective clothing that encompass both basic properties and specified functions.

## 2. Materials and Methods

### 2.1. Materials

Tencel^®^ fibers (Lyocell, Asiatic Fiber Corporation, Taipei, Taiwan) have a length of 51 mm and a fineness of 1.7 dtex. Low-melting polyester (LMPET; Huvis, Seoul, Korea) have a length of 64 mm, a fineness of 4 D, and a melting point of 110 °C. Two types of recycled Kevlar fibers (E. I. du Pont de Nemours and Company, Wilmington, NC, USA) are used, including K129 and K29, which, separately, has an original fineness of 2820D and 1000D and a recycled fineness of 2–3 D, and a length of 50–60 mm. Thermoplastic polyurethanes (TPU; HV-7280EB, Headway Polyurethane Co., Ltd., Hsinchu, Taiwan) has a melt index being 32 g/10 min (measured at 190 °C/8.7 kg). Polyacrylonitrile (PAN; Echo Chemical Co., Ltd., Miaoli, Taiwan) has an Mw being 150,000. *N*,*N*-dimethylformide dimethylformide (DMF; Echo Chemical Co., Ltd., Miaoli, Taiwan) has a concentration ≥98.8%. 

### 2.2. Methods

According to Table 1, nonwoven fabrics, coated nonwoven fabrics, and lay-up compound matrices are separately prepared with the specifications and compositions, and denoted as Tencel and K40 (i.e., two types of nonwoven fabrics), K40/PAN (i.e., the coated nonwoven fabric), and K40/PAN/TPU (i.e., the lay-up compound matrices.)

#### 2.2.1. Preparation of Nonwoven Fabrics

Nonwoven fabrics are produced with different fiber blending ratios, after which LMPET fibers are added for reinforcement, as shown in Table 1. The optimal Tencel/Kevlar nonwoven fabrics are determined according to the results of measurements. Firstly, an automatic hopper opener is used to scatter the fiber lumps and keep them fluffy, and then a card flat is used to mix different fibers. After the breaking and opening, Tencel and Kevlar fibers are separately processed with a cotton comber, a cylinder, a doffer, and then they undergo the carding to form oriented webs that are needle bonded and laminated by a needle punching machine, forming Tencel/Kevlar nonwoven fabrics [25,26,27,28,29]. The needle-bonded rate is 230 needle/min while the needle-bonded depth is 12 mm. Finally, nonwoven fabrics are hot pressed at 160 °C at a rate of 5 rpm/min.

#### 2.2.2. Preparation of PAN Nanofiber Membranes

A magnetic stirrer is used to blend PAN (8 wt%) and DMF until PAN is totally dissolved, forming a yellowish PAN solution. The solution is kept at room temperature for six hours and an ultrasonicator is used to remove bubbles [30,31,32]. Afterwards, an electrospinning machine is used to produce PAN nanofibrous membranes with parameters: a spinning rate of 0.5 mL/h, a voltage of 15 kV, a collection distance of 15 cm, a temperature of 25–30 °C, and a relative humidity of 20%.

#### 2.2.3. Preparation of TPU Breathable and Waterproof Membranes

TPU grains are dissolved into a solution via a solvent, and the TPU solution is smeared over a release paper and dried in an oven, forming the TPU membranes [33].

#### 2.2.4. Preparation of Lay-Up Compound Matrices

**Figure 1:**
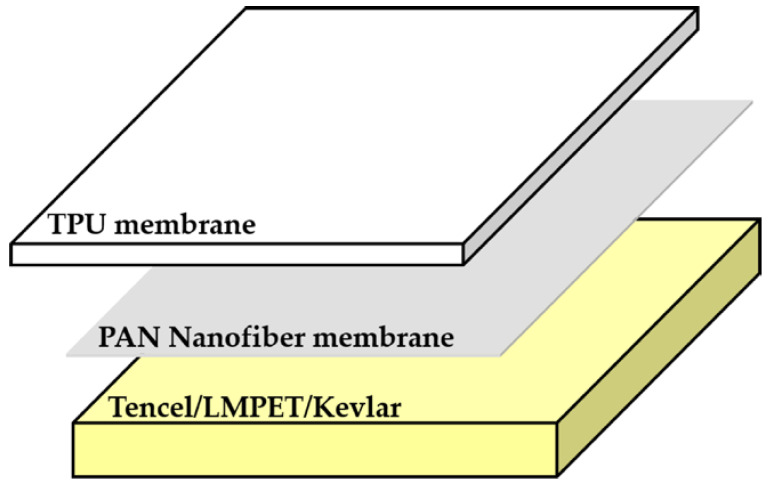
Diagram of the composition of lay-up compound matrices.

Used as the collection board, a Tencel/Kevlar nonwoven fabric is electrospun with a layer of PAN nanofibers (i.e., a PAN nanofibrous membrane), after which a TPU membrane is adhered over the top of the PAN membrane, forming the lay-up compound matrices for medical protective clothing (according to Figure 1). K40/PAN/TPU lay-up compound matrices have a weight per unit area of 70–90 g/m^2^ and a thickness of 1.8–2.0 mm.

### 2.3. Characterizations

#### 2.3.1. Tensile Test

As specified in CNS 12915 test standard, a universal testing machine (HT-2402, Hung Ta Instrument Co., Ltd., Taiwan) is used to measure the tensile properties of samples. The tensile rate is (305 ± 13) mm/min; the sample size is 18 cm × 2.5 cm; and the distance between the fixture is 7.6 cm. Six samples are taken for each specification, individually along the machine direction (MD) and the cross-machine direction (CD) when samples are discharged from the needle punching machine. The collected data are averaged, while the standard errors, as well as the coefficient of variation, are computed.

#### 2.3.2. Tearing Test

As specified in CNS12915 test standard, a universal testing machine (HT-2402, Hung Ta Instrument Co., Ltd., Taichung, Taiwan) is used to measure the tear characteristics of the samples. Five samples are taken along the MD and the CD for each specification, respectively. Samples are a trapezoid with a long base of 150 mm and a short base of 75 mm. The short base is severed in the center perpendicularly with a notch of 1 cm depth. The fixture is used to fasten the two legs of trapezoid. The width of the fixture is 75 mm and the distance of fixture is 25.4 mm. The tearing strength of samples are averaged.

#### 2.3.3. Bursting Test

As specified in CNS 12915 test standard, a universal testing machine (HT-2402, Hung Ta Instrument Co., Ltd., Taichung, Taiwan) is used to measure the bursting strength (N) at a rate being 10 mm/min. Samples have a size of 130 mm × 130 mm.

#### 2.3.4. Puncture Resistance Test

As specified in ASTM F1342-05 test standard, a universal testing machine (HT-2402, Hung Ta Instrument Co., Ltd., Taichung, Taiwan) is used to measure the puncture resistance of samples at a static state. The test rate is (100 ± 10) mm/min and the sample size is 10 cm × 10 cm.

#### 2.3.5. Stiffness Test

As specified in CNS 12915 test standard, the softness (stiffness) of samples is measured and presented with centimeter (cm). The longer the length, the stiffer the samples.

#### 2.3.6. Surface Resistance Test

As specified in BS EN 1149-3:2004 Electrostatic Properties of Protective Clothing (CNS 16081-3 L3279-3), section III: Test Methods for Measurement of Charge Decay, the surface resistance of samples is measured. Samples have a size of 45 mm × 45 mm; the distance between the sample and the detector is (15 ± 1) mm; and the test voltage is 10 kV. Ten samples for each specification are used for the measurement.

#### 2.3.7. Air-Permeable Property Test

As specified in ASTM D0730 test standard, the air-permeability tester (TEXTEST FX3300, TEXTEST Instruments Co., Ltd., Schwerzenbach, Switzerland) is used to measure the air permeability. Samples have a size of 25 cm × 25 cm, and twelve samples for specification are used for the test.

#### 2.3.8. Sub-Micron Particulate Filtration Efficiency

As specified in the protection gear test standard (FTTSF-FP-103), NaCl aerosols with a median particle size (CMD) of (0.075 ± 0.02) um and a geometric standard deviation (GSD) < 1.86 are used in this measurement. The relative humidity is (30 ± 10)% and the temperature is (25 ± 5) °C. The concentration of the aerosol is less than 200 mg/m^3^ and the flux velocity of the aerosol is (32 ± 2) L/min. The required minimal filtration efficiency has to be ≥70%. Six samples for each specification are used for the test. 

#### 2.3.9. Penetration of Synthetic Blood

As specified in CNS14799 test standard, the constant pressure is used to exert the synthetic blood over the protective gear, and it is observed whether the synthetic blood penetrates the samples (75 mm × 75 mm). Synthetic blood (50–55 mL) is infused into the penetration test slot from the upper entrance via a funnel or an injector, after which the subsequent process is observed for five minutes. The measurement is stopped when synthetic blood penetrates the samples.

For clarity and comparison, the measurements have now been summarized in Table 2.

## 3. Results 

### 3.1. Tensile Strength

**Figure 2:**
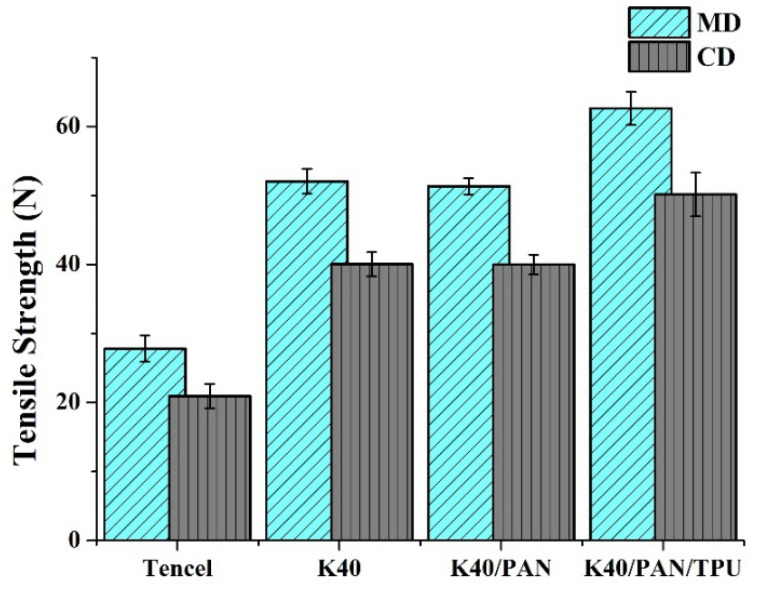
Tensile strength along the MD and CD of Tencel, K40, and K40/PAN/TPU lay-up compound matrices.

The tensile property test is conducted to confirm the tension of materials, one of a series of mechanical characteristics. Hence, the tensile strength test is conducted prior to other mechanical tests. Figure 2 compares the nonwoven fabrics, coated nonwoven fabric, and lay-up compound matrices in terms of tensile strength. The incorporation of Kevlar fibers has a significantly positive influence over tensile strength of the K40 group. Kevlar fibers are more mechanically robust than Tencel fibers, and Kevlar fibers also have a greater amount per unit area when compared to Tencel fibers, which, in turn, facilitates fibers to be better entangled and bonded. Due to hot pressing, the constituent LMPET fibers cause a greater amount of thermal bonding points, preventing the fibers from slippage. The tensile strength of the Tencel group is (27.8 ± 1.8) N along the MD and (20.9 ± 1.7) N along the CD. By contrast, the K40 group comprises 40 wt% of Kevlar fibers and have a higher tensile strength of (52.0 ± 1.7) N along the MD and (40.0 ± 1.7) N along the CD [34].

Nonetheless, coating a PAN nanofibrous membrane does not affect the tensile strength of the K40/PAN group. Compared to Tencel and Kevlar fibers, PAN nanofibers exhibit lower mechanical properties. When PAN is electrospun over nonwoven fabrics, the forming PAN nanofibrous membrane cannot mechanically strengthen the matrices as PAN only provides physical adhesion. With a comparable tensile strength of the Tencel group and K40 group, the K40/PAN group exhibits the tensile strength of (51.3 ± 1.1) N along the MD and (40.0 ± 1.4) N along the CD.

By contrast, the lay-up compound matrices (K40/PAN/TPU group) show a greater tensile strength due to the presence of TPU membranes as the top layer. Similar to the thermal bonding points caused by the heat treatment, the TPU membrane also reinforces the bonding among fibers, and thus has a positive influence over the tensile strength of lay-up compound matrices. The tensile strength is (62.6 ± 2.4) N along the MD and is (50.1 ± 3.1) N along the CD. As for nonwoven fabrics, there is a difference in the tensile strength along the MD and along the CD, which is mainly ascribed to the compliance of fibers.

### 3.2. Tearing Strength

**Figure 3:**
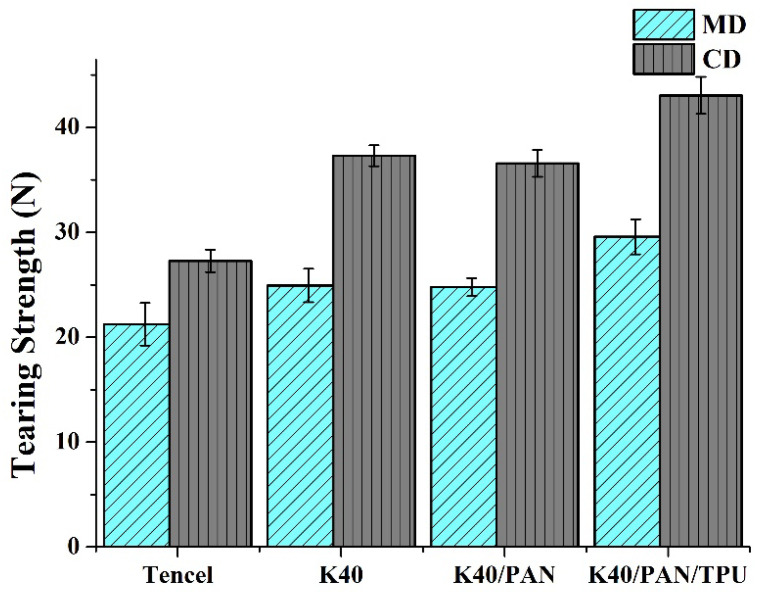
Tearing strength along the MD and CD of Tencel, K40, and K40/PAN/TPU lay-up compound matrices.

In addition to tensile strength, wearable protection gear is also required to be able to resist the damage that is exerted along the notch, which means that tearing strength is another essential attribute. Figure 3 shows the tearing strength of nonwoven fabrics, coated nonwoven fabric, and lay-up compound matrices, where it can be summarized that the tearing strength is improved as a result of the presence of Kevlar fibers and then improved to a greater extent due to the combination of a TPU membrane. TPU membranes supply adhesion that optimizes the thermal bonding points, which, concurrently, makes lay-up compound matrices mechanically robust. The tearing strength along the MD/CD is (21.2 ± 2.0) N/(27.2 ± 1.0) N for the Tencel group, (24.9 ± 1.5) N/(37.3 ± 0.9) N for the K40 group, (24.7 ± 0.8) N/(36.5 ± 1.2) N for the K40/PAN group, and (29.5 ± 1.6) N/(43.0 ± 1.7) N for the K40/PAN/TPU group. Nonwoven fabrics exhibit a marginally increasing trend in the tearing strength along the MD that is precisely the direction of the compliance of nonwoven fabrics. Samples are trimmed with a notch in advance and the tearing force is exerted along the notch, and damage is presented among fibers in the vulnerable direction. Because nonwoven fabrics demonstrate compliance along the MD, fibers guide the spread. A marginal increase in the tearing strength along MD is attributed to thermal bonding points formed by LMPET fibers, instead of Kevlar fibers. In this case, Kevlar fibers merely assist LMPET fibers to form more bonding points per unit area. By contrast, the tearing strength along the CD is dependent on the content of Kevlar fibers. When the tearing strength test is conducted along the CD, there is a greater quantity of horizontally aligned Kevlar fibers that hamper the tearing damage. Meanwhile, the thermosetting behavior of LMPET fibers further advances the difficulty of the tear spreading. As a result, nonwoven fabrics demonstrate a greater tearing strength along the CD.

### 3.3. Bursting Strength

**Figure 4:**
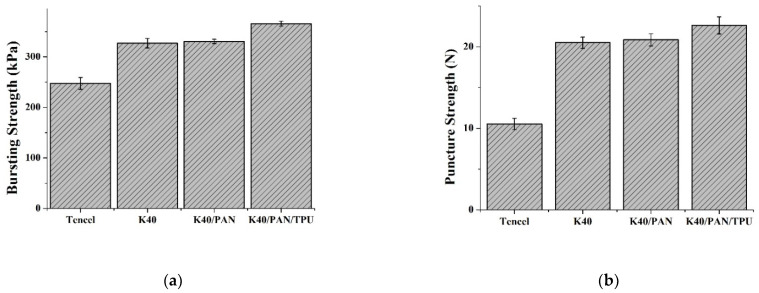
(**a**) Bursting strength and (**b**) static puncture resistance of Tencel, K40, and K40/PAN/TPU lay-up compound matrices.

Bursting strength is an index where fabrics can resist the partial damage caused by a vertical force. When users wear protective gear, they render a force over the elbows, knees, and articulations, which, in turn, deforms these areas. Fibers are broken along the notch, subsequently. Hence, bursting strength and tearing strength share the same trend. Figure 4a shows that the bursting strength of nonwoven fabrics is proportional to the content of Kevlar fibers, and it is once again improved when a TPU membrane is incorporated. The bursting strength is (247.4 ± 11.8) kPa for the Tencel group, (327.0 ± 9.4) kPa for the K40 group, (330.5 ± 4.3) kPa for the K40/PAN, and (365.8 ± 5.0) kPa for the K40/PAN/TPU group. In addition to high mechanical characteristics, Kevlar fibers are also finer than Tencel fibers and thus increase the fabric density, providing nonwoven fabrics with a greater friction force and a greater resistance against the fixture in the bursting strength test. To sum up, the incorporation of Kevlar fibers has a positive influence over the bursting strength of nonwoven fabrics, and the subsequent adhesion of TPU membranes further secures the bonding among fibers for the lay-up compound matrices [35].

### 3.4. Puncture Resistance

The puncture resistance test and the bursting test are similar and both are conducted to measure the resistance in how samples receive and react to the impact of the fixture. The only difference is in the shape of the fixture. The fixture is semicircular in the bursting strength test, while it is needle-like in the puncture resistance test. Hence, in the puncture resistance, the area of thrust surface is rather small with an emphasis on the component force vertically. Figure 4b shows that the K40/PAN/TPU group exhibits an optimal puncture resistance. With a specified basis weight, the quantity of Kevlar fibers is higher than that of Tencel fibers, which is beneficial for fabric density. A greater specific area strengthens the frictional force between fibers and the needle-like fixture [25,33,36]. However, the incorporation of a TPU membrane only improves the adhesion among fibers, as well as prevents fibers from scattering, rather than exerting reinforcement over the component force. Hence, comparing to the bursting strength, the penetration resistance of lay-up compound matrices is not significantly improved. The puncture resistance is (10.5 ± 0.7) N for the Tencel group, (20.5 ± 0.7) N for the K40 group, (20.8 ± 0.7) N for the K40/PAN group, and (22.6 ± 1.0) N for the K40/PAN/TPU group.

### 3.5. Stiffness

**Figure 5:**
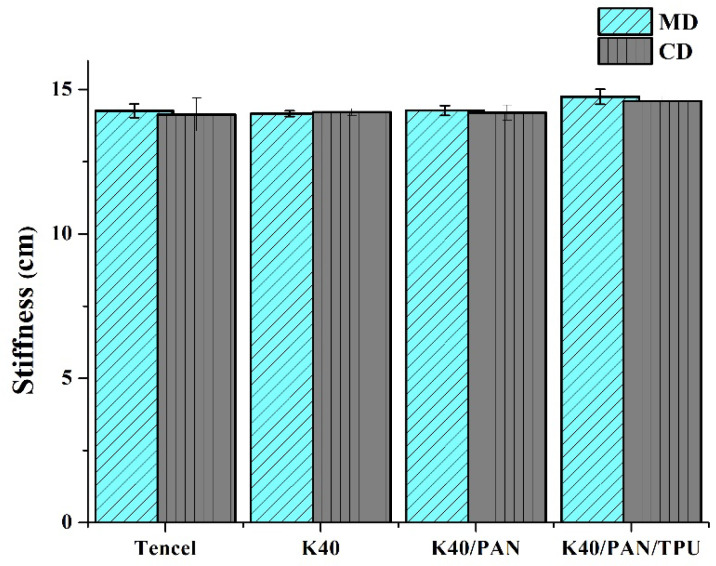
Stiffness along the MD and CD of Tencel, K40, and K40/PAN/TPU lay-up compound matrices.

Protection gear is commonly used by the users for a certain period of time and, therefore, it has to be mechanically robust as well as comfortable, the latter attribute of which is verified by the stiffness measurement. Figure 5 shows the stiffness of nonwoven fabrics, coated nonwoven fabrics, and lay-up compound matrices. Although Tencel fibers are natural fibers that demonstrate a more comfortable texture than Kevlar fibers, the nonwoven fabrics made in this study are incorporated with LMPET fibers that significantly increase the stiffness after hot pressing. In sum, the Tencel group and the K40 group exhibit comparable stiffness, and the K40/PAN group also has similar stiffness because the constituent PAN membrane only possesses a rather low thickness. Only the K40/PAN/TPU group demonstrates a slightly higher stiffness that is ascribed to the adhesive agent [37]. The stiffness along the MD/CD is separately (14.2 ± 0.2) cm/(14.1 ± 0.5) cm for the Tencel group, (14.1 ± 0.3) cm/(14.2 ± 0.1) cm for the K40 group, (14.2 ±0.1) cm/(14.2 ± 0.2) cm for the K40/PAN group, and (14.7 ± 0.2) cm/(14.6 ± 0.1) cm for the K40/PAN/TPU group.

### 3.6. Surface Resistance

**Figure 6:**
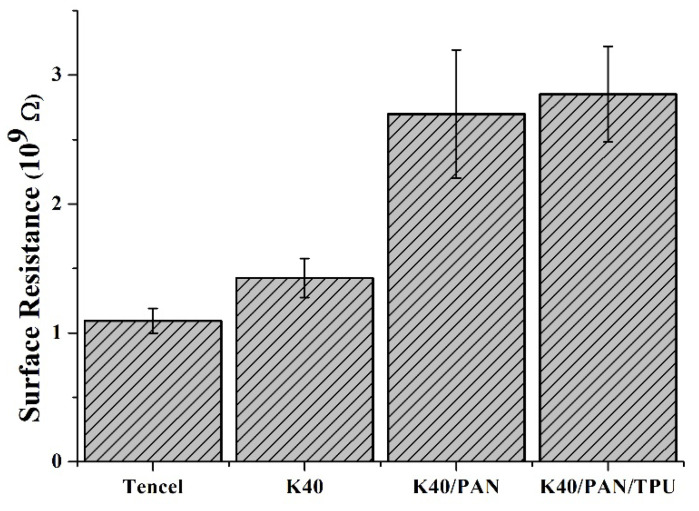
Surface resistance of Tencel, K40, and K40/PAN/TPU lay-up compound matrices.

The surface resistance measurement is conducted to examine the electrostatic properties of matrices for medical protective clothing. On one hand, the surface resistance of matrices prevents medical instruments from electromagnetic interferences. On the other hand, the surface resistance of matrices reduces the massive amount of electrostatic interference, thereby keeps the users comfortable. Figure 6 shows that the surface resistance is (1.09 × 10^9^ ± 0.09 × 10^9^) Ω for the Tencel group, (1.42 × 10^9^ ± 0.15 × 10^9^) Ω for the K40 group, (2.69 × 10^9^ ± 0.49 × 10^9^) Ω for the K40/PAN group, and (2.85 × 10^9^ ± 0.37 × 10^9^) Ω for the K40/PAN/TPU group. Tencel fibers are composed of comparatively more O-H groups and thus have a low resistor value. In addition to Kevlar fibers, a combination of lamination layers also improves the surface resistance and the hydrophobic bond.Although the surface resistance is constantly increased due to the composition, it is still within an appropriate application range.

### 3.7. Air-Permeable Property

**Figure 7:**
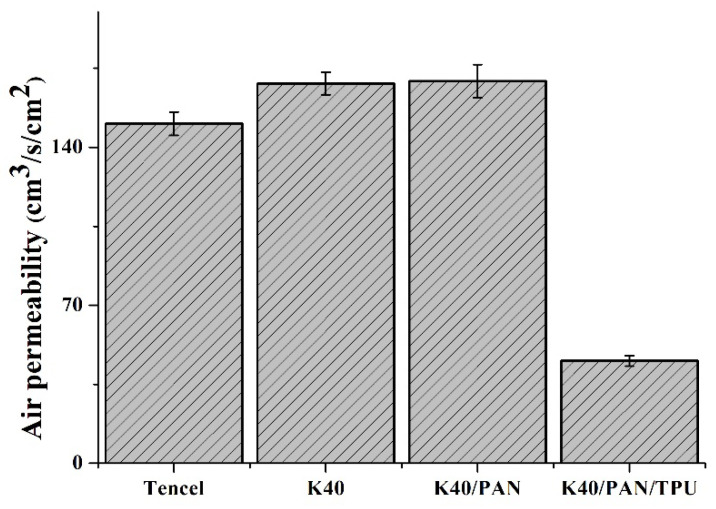
Air permeability of Tencel, K40, and K40/PAN/TPU lay-up compound matrices.

Referring to the permeability of air through the materials, an appropriate air permeability indicates that the protection gear is comfortable for the users, just like the demands of stiffness and surface resistance. Figure 7 shows an air permeability of (150.4 ± 5.1) cm^3^/s/cm^2^ for the Tencel group, (168.2 ± 5.0) cm^3^/s/cm^2^ for the K40 group, (169.3 ± 7.2) cm^3^/s/cm^2^ for the K40/PAN group, and (45.4 ± 2.3) cm^3^/s/cm^2^ for the K40/PAN/TPU group. Tencel fibers are characterized by high moisture regain and fluffy/draping textures because they are crimped. The crimped attribute provides Tencel fibers with a certain degree of air permeability, whereas a high moisture regain renders Tencel fibers with hygroscopic expansion, which subsequently increases the fiber diameter and the bending degree. Eventually Tencel fibers are aligned more compactly, which hinders the air flux. As a result, Tencel nonwoven fabrics demonstrate a lower air permeability than Kevlar nonwoven fabrics. Kevlar fibers have a sleek surface and a low flexibility, which facilitates the ventilation of air flux. Nonetheless, the density of Kevlar fibers also increases the amount of thermal bonding points formed by LMPET fibers, and therefore, a rise in the Kevlar fibers does not change the air permeability. Similarly, the presence of PAN nanofibrous membranes does not interfere with air permeability because nanofibers are evenly distributed and the PAN nanofibrous membranes are porous, which does not hamper the air flux. As far as lay-up compound matrices are concerned, the air permeability is largely decreased after TPU membranes are adhered. There are two major factors. One is that TPU membranes do not possess as large pore sizes as nonwoven fabrics do, and the other is that the adhesive agent between a TPU membrane and a nonwoven fabric seals the majority of pores. As a result, the K40/PAN/TPU group demonstrates a compromised air permeability [38].

### 3.8. Sub-Micron Particulate Filtration Efficiency

**Figure 8:**
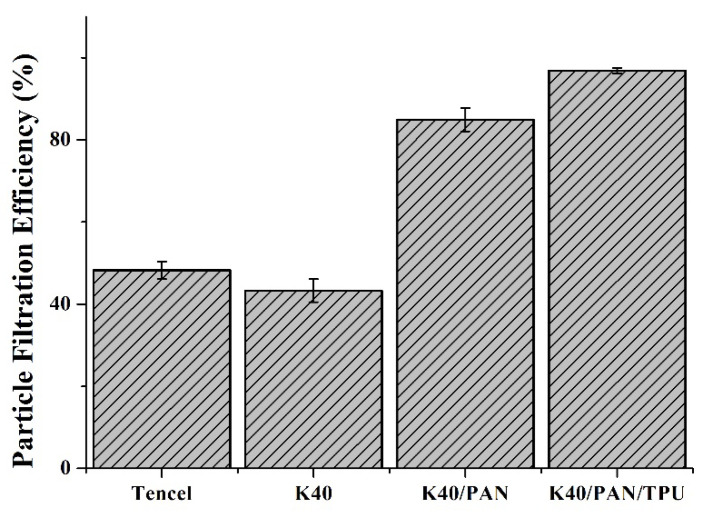
Sub-micron particulate filtration efficiency of Tencel, K40, and K40/PAN/TPU lay-up compound matrices.

**Figure 9:**
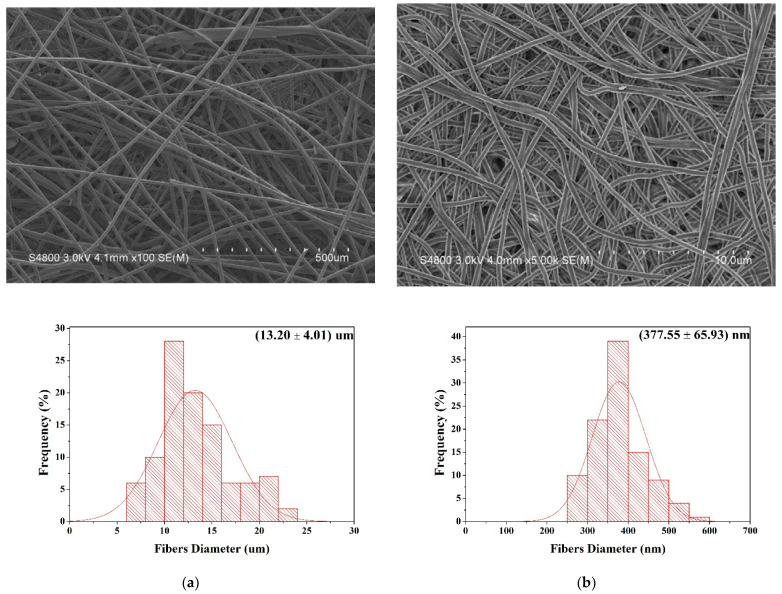
SEM images of (**a**) K40: ×100 (**b**) K40/PAN: ×5 K, showing the fiber morphology and diameter.

Sub-micron particulate filtration efficiency serves as one index that stops the blood penetration and microorganisms. Generally, filter media is mainly used to block the particles of a fluid while strengthening the collision between particles and the media. In particular, porosity and air permeability are two important functions that are required when fabrics are used as a filter. Besides, fabric filters can also acquire filtration efficiency when they are loaded with electric charge. Then they can adsorb particles with their electrostatic force or via the electrostatic charging. However, the design of this study stresses the application as medical protection gear commonly contacts liquids, e.g., water and bodily solution, which compromise the electrostatic force. Therefore, physical resistance of suspensive particles from the air is emphasized in this study, and fiber structure is changed to satisfy the demands of medical protection gear. A high filtration efficiency thus guarantees the quality of protective clothing. Figure 8 shows that the K40 group exhibits a decreasing trend in the filtration efficiency compared to the Tencel group, and the filtration efficiency decreases from (48.2 ± 2.1)% to (43.3 ± 2.8)%. According to the air permeability in Figure 7, the air permeability of the K40 group is higher than that of the Tencel group, which allows the particle to penetrate the K40 nonwoven fabrics with the air. Coated with a PAN membrane, the K40/PAN group exhibits a filtration efficiency that is highly improved to (84.9 ± 2.8)%. The PAN nanofibers are evenly distributed and the PAN membranes thus possess a microporous structure that can effectively block the sub-micron particles (Figure 9 shows the fiber morphology and diameter of different samples). Finally, the lay-up compound matrices demonstrate a strengthened filtration efficiency of (98.1 ± 0.8)% because of the adhered TPU membrane, suggesting an effective filtration that meets the application standard for sub-micron penetration.

### 3.9. Penetration of Synthetic Blood

After protection gear achieves specified mechanical properties and comfortable texture, the application ends and the functions are considered. As for medical protection gear, the prevention of microbes is a priority. Table 3 indicates that the Tencel group and the K40 group allow the penetration of synthetic blood in an atmosphere status and a negative pressure status. Synthetic blood can penetrate both groups because nonwoven fabrics are porous with large pore sizes. Moreover, the K40/PAN group shows an unpenetrated status when in the atmosphere status for the PAN nanofibrous membrane with a fine porosity and hydrophobicity is able to prevent the blood from penetrating the sample. Regardless of whether it is in an atmosphere status or a negative pressure status, K40/PAN/TPU lay-up compound matrices can block the synthetic blood due to the incorporation of a waterproof TPU membrane. Even when the surface is stained, the K40/PAN/TPU group can still protect human skin from being penetrated with foreign substances.

Note. “—” means that synthetic blood can penetrate the sample while “√” means that synthetic blood fails in penetrating the sample.

### 3.10. Comparison of Test Data and Regulated Standards

Table 4 shows all of the test data collected from each measurement where the comparisons of matrices are proceeded as related to the measurement and the test standard.

1. As specified in the test standard, the tensile test along the MD is required to be ≥50 N and that along the CD needs to be ≥40 N. The Tencel group fails in this standard as the tensile strength is (27.8 ± 1.8) N (MD) and (20.9 ± 1.7) N (CD). The K40 group meets the standard as the tensile strength is (52.0 ± 1.7) N (MD) and (40.0 ± 1.7) N (CD). Similarly, both the K40/PAN and the K40/PAN/TPU groups also meet the requirement of the standard.
2. As specified in the test standard, the tearing strength along the MD/CD should be ≥20 N. The Tencel group meets the test standard for the tearing strength along the MD and CD is separately (21.2 ± 2.0) N and (27.2 ± 1.0) N, so do all of the other matrices, including the K40, K40/PAN, and K40/PAN/TPU groups.
3. As specified in the test standard, the bursting strength should be ≥200 kPa, which is acquired by all of the matrices, including the Tencel, K40, K40/PAN, and K40/PAN/TPU groups. The bursting strength of the Tencel group is (247.4 ± 11.8) kPa.
4. As specified in the test standard, the puncture resistance should be ≥20 N. Except for the Tencel group, all of the other groups outperform the Tencel group and meet the standard. The K40 group exhibits a puncture resistance of (20.5 ± 0.7) N.
5. As specified in the test standard, the stiffness should be ≤15 cm. The Tencel, K40, K40/PAN, and K40/PAN/TPU groups do not differ much in stiffness. Although hot pressing renders the nonwoven fabrics with a higher stiffness, which does not exclude the K40/PAN and K40/PAN/TPU groups from showing a qualified stiffness, which is ascribed to a lower thickness of both types of nonwoven fabrics.
6. As specified in the test standard, the surface resistance should be as 1 × 10^9^ ≤ Ω ≤ 1 × 10^12^. The Tencel, K40, K40/PAN, and K40/PAN/TPU groups exhibit standard surface resistance.
7. As specified in the test standard, the air permeability should be ≥40 cm^3^/s/cm^2^. The Tencel group exhibits the highest air permeability, followed by the K40, K40/PAN, and then K40/PAN/TPU groups. Despite the air permeability being (45.4 ± 2.3) cm^3^/s/cm^2^, the K40/PAN/TPU group still meet the standard.
8. As specified in the test standard, the sub-micron particulate filtration efficiency should be ≥70%. Both the K40/PAN and K40/PAN/TPU groups meet the standard, and the latter of which exhibits a sub-micron particulate filtration efficiency of (84.9 ± 2.8)%.
9. As specified in the test standard for penetration of synthetic blood, it is a must that samples are completely blood proof. Therefore, only the K40/PAN/TPU group meets the test standard either in the atmospheric pressure or negative pressure.

To sum up, the K40 group can satisfy the basic protection requests. In addition to a dust-proof property, K40/PAN can also satisfy mechanical properties, comfortable texture, and sub-micron particulate filtration efficiency (84.9 ± 2.8)%. Eventually, it is only the K40/PAN/TPU group that can satisfy all of the aforementioned properties as well as the synthetic blood-proof one.

## 4. Conclusions

In this study, the nonwoven process, electrostatic spinning, and film forming technology are employed, which successfully produce lay-up compound matrices that have excellent mechanical properties, comfort, and functions, and are thus suitable for medical protective clothing. In the comparison of two types of nonwoven fabrics—the Tencel group and the K40 group—, the K40 group outperforms the Tencel group in terms of mechanical performances, the results of which are presented in the order of Tencel group/K40 group, as follows: the tensile strength is (27.8 ± 1.8) N (MD)/(62.6 ± 2.4) N (MD) as well as (20.9 ± 1.7) N (CD)/(50.1 ± 3.1) N (CD); the tearing strength is (21.2 ± 2.0) N(MD)/(29.5 ± 1.6) N (MD) as well as (27.2 ± 1.0) N (CD)/(43.0 ± 1.7) N(CD); the bursting strength is (247.4 ± 11.8) kPa/(365.8 ± 5.0) kPa; and the puncture strength is (10.5 ± 0.7) N/(22.6 ± 1.0) N. Notably, the nonwoven fabrics are mechanically strengthened because of the incorporation of Kevlar fibers, and the subsequent lamination process further improves the reinforcement. When it comes to the comfort texture of the Tencel group/K40 group, the stiffness is (14.2 ± 0.2) cm (MD)/(14.7 ± 0.2) cm (MD) as well as (14.1 ± 0.5) cm (CD)/(14.6 ± 0.1) cm(CD); the surface resistance is (1.09 × 10^9^ ± 0.09 × 10^9^) Ω/(2.85 × 10^9^ ± 0.37 × 10^9^) Ω; and the air-permeable property is (150.4 ± 5.1) cm^3^/s/cm^2^ / (45.4 ± 2.3) cm^3^/s/cm^2^. Moreover, the nonwoven fabrics also exhibit a sub-micron particulate filtration efficiency that is higher than 98%. Finally, the penetration of synthetic blood is absent in the K40/PAN/TPU lay-up compound matrices. Consisting of lamination layers, the matrices appear comparatively stiffer at a cost of the loss of air permeability, whereas the lay-up compound matrices obtain remarkably improved sub-micron particulate filtration efficiency and penetration of synthetic blood. In addition, the test results indicate that the use of Kevlar fibers provides medical protective clothing with mechanical properties with additionally preserved comfort, and this study establishes a fundamental structure design as well as a highly feasible application range.

## Figures and Tables

**Table 1:** Specifications and denotations of nonwoven fabrics and lay-up compound matrices.

	Tencel (wt%)	LMPET (wt%)	Kevlar (wt%)	PAN Nanofiber Membrane	TPU Membrane
Tencel	70	30	-	-	-
K40	30	30	40	-	-
K40/PAN	30	30	40	√	-
K40/PAN/TPU	30	30	40	√	√

**Table 2:** Specifications of samples required by test standard.

Testing	Test Criterion	Sample Size (cm^2^)	Standard
Tensile strength	CNS 12915	18 × 2.5	≥50 N(MD)
			≥40 N(CD)
Tearing strength	CNS 12915	15 × 7.5	≥20 N(MD/CD)
Bursting strength	CNS 12915	13 × 13	≥200 kPa
Puncture strength	ASTM F1342-05	10 × 10	≥20 N
Stiffness	CNS 12915	15 × 2.5	≤15 cm
Surface resistance	BS EN 1149-3:2004	4.5 × 4.5	1 × 10^9^ ≤ Ω ≤ 1×10^12^
Air-permeable property	ASTM D0730	25 × 25	≥40 cm^3^/s/cm^2^
Sub-micron particulate filtration efficiency	FTTS-FP-103	15 × 15	≥70%
Penetration of synthetic blood	CNS14799	7.5 × 7.5	nonpenetration

**Table 3:** Penetration of synthetic blood for Tencel, K40, and K40/PAN/TPU lay-up compound matrices at atmospheric pressure and negative pressure.

	Tencel	K40	K40/PAN	K40/PAN/TPU
**Atmospheric pressure**	—	—	√	√
**Negative pressure**	—	—	—	√

Note. “—“ means that synthetic blood can penetrate the sample while “√“ means that synthetic blood fails in penetrating the sample.

**Table 4:** Data of mechanical properties (N ≥ 6).

Testing/Sample		Tencel	K40	K40/PAN	K40/PAN/TPU
Tensile strength (N)	MD	27.8 ± 1.8	52.0 ± 1.7	51.3 ± 1.1	62.6 ± 2.4
	CD	20.9 ± 1.7	40.0 ± 1.7	40.0 ± 1.4	50.1 ± 3.1
Tearing strength (N)	MD	21.2 ± 2.0	24.9 ± 1.5	24.7 ± 0.8	29.5 ± 1.6
	CD	27.2 ± 1.0	37.3 ± 0.9	36.25 ± 1.2	43.0 ± 1.7
Bursting strength (kPa)		247.4 ± 11.8	327.0 ± 9.4	330.5 ± 4.3	365.8 ± 5.0
Puncture strength (N)		10.5 ± 0.7	20.5 ± 0.7	20.8 ± 0.7	22.6 ± 1.0
Stiffness (cm)	MD	14.2 ± 0.2	14.1 ± 0.1	14.2 ± 0.1	14.7 ± 0.2
	CD	14.1 ± 0.5	14.2 ± 0.1	14.2 ± 0.2	14.6 ± 0.1
Surface resistance (10^9^ Ω)		1.09 ± 0.09	1.42 ± 0.15	2.69 ± 0.49	2.85 ± 0.37
Air-permeable property (cm^3^/s/cm^2^)		150.4 ± 5.1	168.2 ± 5.0	169.3 ± 7.2	45.4 ± 2.3
Sub-micron particulate filtration efficiency (%)		48.2 ± 2.1	43.3 ± 2.8	84.9 ± 2.8	98.1 ± 0.8

## Data Availability

All data relevant to the study are included in the article.
